# Establishment of a 10-Plex Quantitative Fluorescent-PCR Assay for Rapid Diagnosis of Sex Chromosome Aneuploidies

**DOI:** 10.1371/journal.pone.0106307

**Published:** 2014-09-10

**Authors:** Xingmei Xie, Qiaoyi Liang

**Affiliations:** 1 Prenatal Diagnostic Center, Guangzhou Women and Children’s Medical Center, Guangzhou, China; 2 Institute of Digestive Disease and Department of Medicine and Therapeutics, State Key Laboratory of Digestive Disease, Li Ka Shing Institute of Health Sciences, CUHK Shenzhen Research Institute, The Chinese University of Hong Kong, Hong Kong; Sanjay Gandhi Medical Institute, India

## Abstract

Sex chromosome aneuploidies occur commonly in the general population, with an incidence of 1 in 400 newborns. However, no tests specifically targeting sex chromosomes have been carried out in prenatal diagnosis or newborn screening, resulting in late recognition of these diseases. In this study, a rapid diagnostic method for sex chromosome aneuploidies was established using Quantitative Fluorescent-PCR (QF-PCR). Ten markers were included in one multiplex QF-PCR assay, including two sex determination genes (AMXY and SRY), five X-linked short tandem repeats (STRs; DXS1053, DXS981, DXS6809, DXS1187, and DXS8377), one X/Y-common STR (X22), and two autosomal STRs (D13S305 and D21S11). Retrospective tests of 70 cases with known cytogenetic results indicated that the 10-plex QF-PCR assay could well determine sex chromosome copy numbers by both allelic peak numbers and a sex chromosome dosage calculation with the autosomal STRs as internal controls. Prospective comparison with cytogenetic karyotyping on 534 cases confirmed that the 10-plex QF-PCR assay could be well employed for sex chromosome aneuploidy diagnosis in at least the Chinese Han population. This is the first QF-PCR test for the diagnosis of sex chromosome aneuploidies in the Chinese population. This test is superior to previous designs by including up to 8 sex-linked markers covering different parts of sex chromosomes as well as employing internal controls for copy number dosage calculation in a single PCR reaction. Due to simple technique and data analysis, as well as easy implementation within routine clinical services, this method is of great clinical application value and could be widely applied.

## Introduction

Sex chromosome aneuploidies are the most common disorder of sex chromosomes in humans, with an incidence of 1 in 400 newborns [1]. Although sex chromosome aneuploidies include a variety of abnormalities of the sex chromosomes, the most commonly occurring sex chromosome aneuploidies involve the deletion (45, X) or addition (47, XXY, 47, XYY, 47, XXX) of an X or Y chromosome. Turner syndrome (45, X) occurs in about 1 per 2,000 live born girls [2]. Klinefelter syndrome (47, XXY) occurs in approximately 1.20 per 1,000 live born male births [3], being the most common genetic cause of severe male factor infertility. With less observable phenotypes, 47, XYY and 47, XXX karyotypes occur in high incidences, with 1 per 1,000 male births and 1 per 1,000 female births respectively. Most autosomal aneuploidies are naturally miscarried before the time of prenatal testing or birth. In contrast, sex chromosomal rearrangements abort at a lower rate [4]. Diagnosis of sex chromosomal aneuploidies is not routinely carried out prenatally or at birth, and most cases are not diagnosed until the newborns/children show related syndromes. Among these conditions, only Turner syndrome results in an easily identifiable physical phenotype, but the median age at diagnosis is 15 years old [5].

The gold standard diagnostic test for prenatal detection of chromosomal aneuploidies is karyotyping analysis of cultured cells obtained from amniotic fluid, chorionic villus sampling, or cord blood. It is capable of detecting all kinds of chromosomal aneuploidies. However, karyotyping has several defects which prohibit its wide use in prenatal diagnosis, including a need for skillful technicians, relatively low success rate even when performed by skillful technicians, time-consuming (with an average reporting time of 14 working days) and cost-consuming. In countries of large populations like China, where the birth control policy is implemented, prenatal diagnosis is of great importance to both the society and families. However, a severe lack of eligible karyotypitsts, together with defects of karyotyping, contributes to an urgent need for developing a rapid and less cost-consuming method to detect chromosome aneuploidies, applicable for at least the Chinese population.

The rapid prenatal diagnosis method by quantitative fluorescence PCR (QF-PCR) has been widely applied since it was introduced. Requiring only simple PCR machines and first generation sequence detectors, QF-PCR could easily be applied in clinical practice by trained technicians. In this study, we developed a 10-plex QF-PCR assay for rapid prenatal diagnosis of sex chromosome aneuploidies. Besides including more X/Y-linked markers, we also included two autosomal STRs in the same PCR reaction system as internal controls for sex chromosome dosage determination. With experimental and analytical procedures well optimized, we further tested this platform both retrospectively and prospectively in comparison with cytogenetic karyotyping and confirmed its clinical application potentials in at least the Chinese Han population.

## Results

### Design of the 10-plex QF-PCR assay

**Figure 1:**
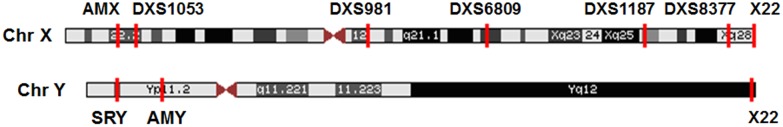
Graphic scheme of the chromosomal localization of the eight sex chromosomal markers used in the newly designed multiplex PCR assay. The markers were located according to Human Genome Browser – hg38 assembly.

In order to establish a multiplex PCR assay with all primer pairs working compatibly with each other, primers were designed using similar criteria for primers and amplicons (such as Tm, GC content), with expected amplicon sizes not overlapping and fluorescent dyes staggered. Short tandem repeat (STR) markers were carefully selected so as to cover different parts of the chromosomes. Five X-linked STRs distributed on both the short and long arms, including DXS1053, DXS981, DXS6809, DXS1187 and DXS8377, the STR located on both the X and Y chromosomes (X22), the modified amelogenin non-polymorphic marker AMXY, and the non-polymorphic sex-determining region of the Y chromosome (SRY) were used for assessing the number of sex chromosomes as well as for sex determination **(** **Figure 1** **)**. Two additional autosomal STRs, D13S305 on chromosome 13 and D21S11 on chromosome 21, were used as internal controls for quantification of the number of sex chromosomes. The resulting 10 primer pairs were accommodated in a single 10-plex PCR system, producing PCR products distributed evenly within the range of 100 to 500 bp. Two different fluorescent dyes were adequate to distinguish adjacent targets. The nucleotide sequences and fluorescent labeling information are listed in **Table 1**, with optimized primer concentrations indicated.

**Table 1:** Marker and primer composition of the new 10-plex assay for the detection of sex chromosome aneuploidies.

STR markers	Primer	5′ Reporter	Nucleotide sequence (5′->3′)	Concentration/µM	
AMXY	F	FAM	CCCTGGGCTCTGTAAAGAATAGTG	0.1	
	R		ATCAGAGCTTAAACTGGGAAGCTG		
DXS1187	F	FAM	TGGAGAAAGTCACTGAACAGAGGA	0.15	
	R		GGCACCTTTCAGCTACTCAATGA		
DXS8377	F	FAM	CCACTTCATGGCTTACCACAGA	0.15	
	R		TTTTGCTCCTTCGTTCCCTG		
SRY	F	HEX	AGTAAAGGCAACGTCCAGGAT	0.2	
	R		TTCCGACGAGGTCGATACTTA		
DXS6809	F	FAM	GGAGGACCATGTTTCACTGGA	0.2	
	R		AGCTCAGGAATACTGAGGGCAT		
DXS981	F	HEX	CATGGTTCTCCTTGTGGCCT	0.3	
	R		TCTCCAGCACCCAAGGAAGT		
DX1053	F	FAM	CTCCATTTGTGCCCTCACTGA	0.3	
	R		GCCTCCACACCTTTGCTTGT		
D13S305	F	FAM	GCCACCTCGAAATCCTAGGC	0.3	
	R		TGTCGTTACGAATGAATGGAAGG		
X22	F	HEX	GTGATTCTGCCAGGATGCAA	0.4	
	R		CCAAGGTGCCTGGAAGAGG		
D21S11	F	FAM	TGGCCTCAAACTGAAGGTTACA	0.4	
	R		AGTGTCTGGCACCCAGTAAAAAA		

### Testing of the new 10-plex QF-PCR assay using samples of known karyotyping results

**Figure 2:**
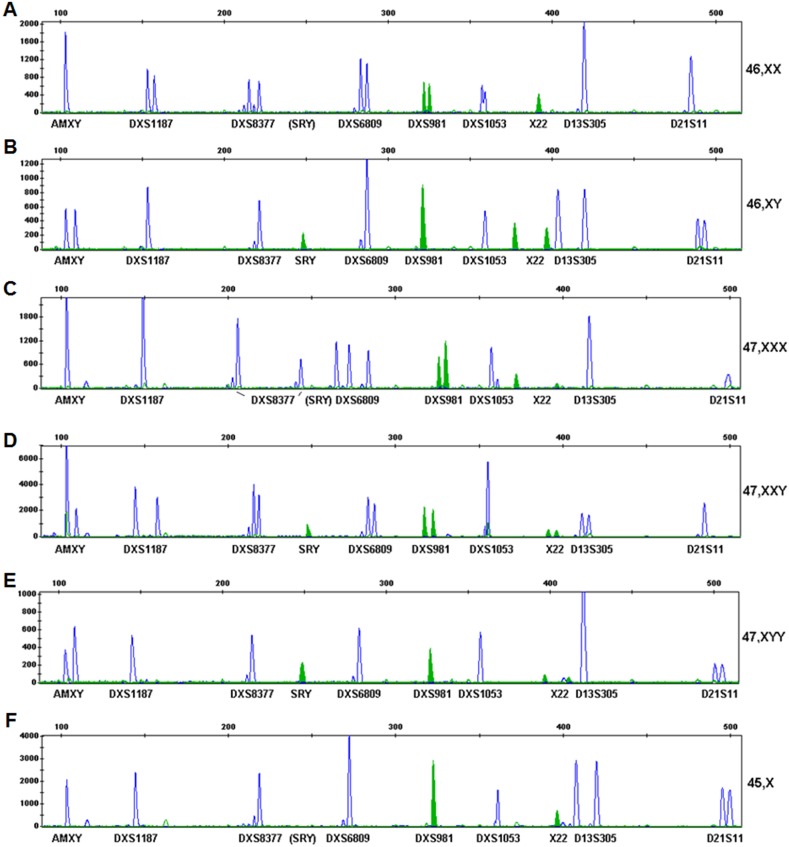
Representative examples for a normal female (A), a normal male (B), a trisomy X (47, XXX) (C), a Klinefelter syndrome (47, XXY) (D), a Jacob’s syndrome (47, XYY) (E) and a Turner syndrome (45, X) (F) obtained through the 10-plex QF-PCR assay. The results are produced using the GeneMapper software, showing full ranges and all sizes of the detected peaks on the vertical and horizontal axes respectively for intuitive intra-sample visual comparison of the peaks. Fragment sizes are shown in bp on the horizontal axis. Arbitrary fluorescence units are shown on the vertical axis.

A total of 50 samples with normal karyotypes (46, XX or 46, XY) and 20 samples with Turner syndrome (45, X), 5 cases of 47, XXY, 5 case of 47, XYY, 5 cases of 47, XXX confirmed by karyotype analysis were subjected to QF-PCR assessment using the new 10-plex QF-PCR assay. The QF-PCR results were informative for all samples tested. Chromatographs led to ‘normal’ diagnosis for all normal samples, with at least one informative X-linked STR with a ratio of 1∶1 for normal female samples **(** **Figure 2A** **)** or all single peak for X-linked STRs (DXS1187, DXS8377, DXS6809, DXS981 and DXS1053), a single peak or two equal peaks for the X/Y-common STR X22, and two equal peaks for AMXY (AMX and AMY) plus an SRY peak for normal male samples **(** **Figure 2B** **)**.

Chromatographs led to ‘abnormal’ diagnosis for all abnormal samples tested. In cases of pure 47, XXX karyotypes, the 10-plex QF-PCR assay showed one peak AMXY (AMX, without AMY), no peak for SRY, single peaks or abnormal peaks of two with a ratio of 1∶2 (2∶1) or three peaks for X/Y-linked STRs **(** **Figure 2C** **)**. In cases of pure 47, XXY karyotypes, two peaks for AMXY with a ratio of 2∶1 (AMX:AMY), one peak for SRY, single peaks or two peaks with a ratio of 1∶1 for all X-linked STRs, and a single peak or two peaks with a ratio of 1∶2 (2∶1) or three peaks for the X/Y common STR X22 were obtained by the 10-plex QF-PCR **(** **Figure 2D** **)**. In cases of pure 47, XYY karyotypes, the 10-plex QF-PCR assay showed two peaks for AMXY with a ratio of 1∶2 (AMX:AMY), one peak for SRY, single peaks for all X-linked STRs, and a single peak or two peaks with a ratio of 1∶2 (2∶1) or three peaks for X22 **(** **Figure 2E** **)**. QF-PCR chromatographs showed single peaks for all X-linked STRs, X22 and AMXY for confirmed Turner samples **(** **Figure 2F** **)**.

### Dosage quantitation of sex chromosomes using two autosomal internal controls

**Figure 3:**
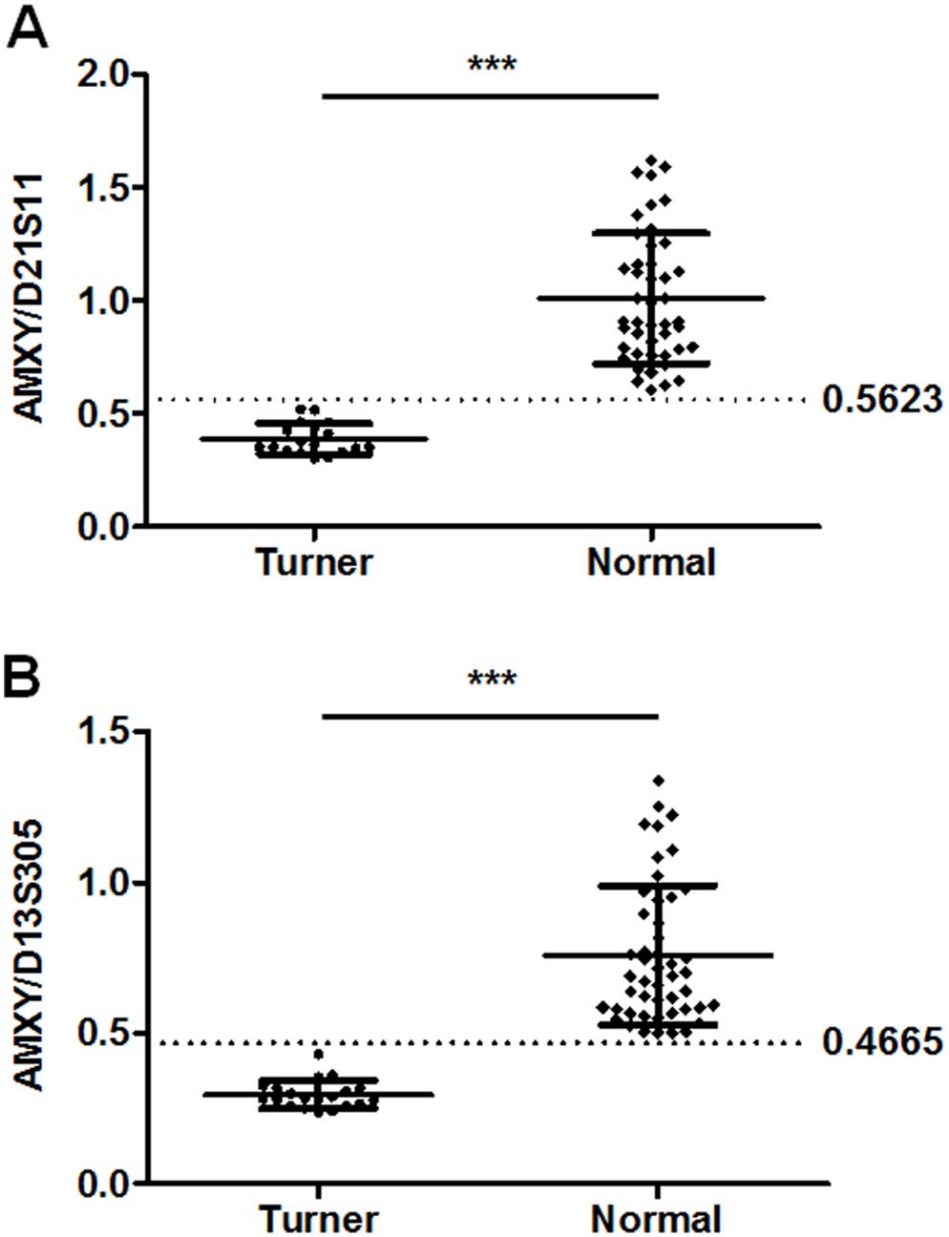
Comparison of peak ratios of AMXY to autosomal internal controls ((A) D21S11 and (B) D13S305) between confirmed Tuner samples and normal samples. Unpaired t tests were used. ***P<0.0001. Dash lines showed the cutoff values of AMXY/D21S11 (0.55) and AMXY/D13S305 (0.5), which would be used as the criteria for determination of Turner syndromes when suspected cases show single peaks for all X-linked markers and no peaks for AMY and SRY.

In order to test the dosage determination capacity of the 10-plex assay, we calculated the peak area ratios of AMXY/D21S11 and AMXY/D13S305 for all samples with known karyotyping results (50 normal and 20 Turner). Samples of Turner syndrome showed AMXY/D21S11 ratios of 0.39±0.07, while normal samples showed ratios of 1.01±0.29 **(** **Figure 3A** **)**. Unpaired *t* test indicated significant difference in AMXY/D21S11 ratios between the Turner and normal groups (*P*<0.0001). Samples with Turner syndrome showed AMXY/D13S305 ratios of 0.30±0.05, while normal samples showed ratios of 0.76±0.23 **(** **Figure 3B** **)**. Unpaired *t* test also indicated significant difference in AMXY/D13S305 ratios between the Turner and normal groups (*P*<0.0001). More importantly, no overlap was observed for the two ratios between Turner and normal groups. Receiver operating curve analysis showed areas under the curve were 1 for both ratios (*P*<0.0001). Best cut-off values that maximizes (sensitivity + specificity), i.e. AMXY/D21S11 = 0.5623 and AMXY/D13S305 = 0.4665, could both discriminate Tuner and normal individuals at 100% sensitivity and 100% specificity. Therefore, when it came to suspected cases of Turner syndrome with single peaks for all X-linked markers and the XY-marker X22, and no peaks for AMY and SRY, AMXY/D21S11<0.5623 and AMXY/D13S305<0.4665 would be the criteria for determination of Turner syndromes (Table S1).

### Application of the new 10-plex QF-PCR assay in diagnosis

**Figure 4:**
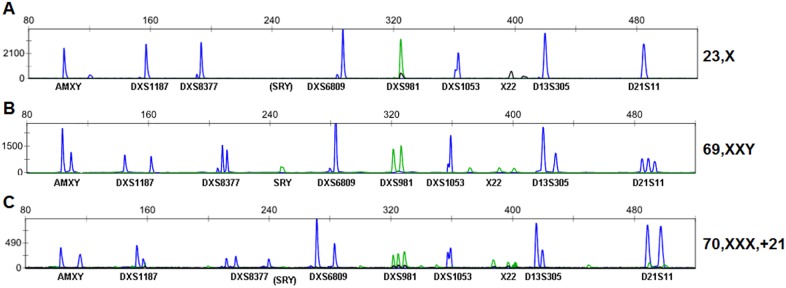
Capillary electrophoresis results for three samples with sex chromosomal aneuploidies identified in the prospective examination, including (A) a suspected 23, X finally confirmed by karyotyping, (B) a case of triploidy ‘69, XXX’, and (C) a case of triploidy with an additional chromosome 21 (70, XXX,+21).

To test the clinical application value of the new 10-plex QF-PCR assay, a total of 534 samples were recruited from patients undergoing prenatal diagnosis, including 55 CV samples from spontaneous miscarriages to increase samples with suspected abnormal karyotypes. QF-PCR assays with the 10-plex PCR system and conventional cytogenetic analysis were carried out at the same time and results were then compared. Cytogenetic analysis successfully produced results for 527 (98.7%) samples tested, while QF-PCR successfully produced analytical results for 99.6% (532/534) samples. The only two samples which failed to produce confirmative results by QF-PCR included one sample showing mosaicism that might be due to maternal DNA contamination, and the other one of ‘23, X’ confirmed to be by karyotyping. The one ‘23, X’ showed no peak for SRY and single peak for all other markers, but ratios of AMXY/D21S11 (0.6728) and AMXY/D13S305 (0.5592) within ‘normal’ ratio ranges (**Figure 4A**). Two triploid cases were found by QF-PCR and further confirmed by karyotyping (**Figures 4B and 4C**). For samples with both successful cytogenetic and QF-PCR analyses, results were consistent in the determination of sex chromosome numbers by the two methods. Diagnostic results by QF-PCR and karyotyping were summarized in **Table 2**.

**Table 2:** Summary of diagnostic results by QF-PCR and karyotyping.

	Amniotic Fluid		Cord Blood		Chorionic Villi-1		Chorionic Villi-2		Total	
sample size	413		39		27		55		534	
Method	QF-PCR	Karyotyping	QF-PCR	Karyotyping	QF-PCR	Karyotyping	QF-PCR	Karyotyping	QF-PCR	Karyotyping
Informative	413 (100%)	410 (99.3%)	39 (100%)	39 (100%)	26 (96.3%)	23 (85.2%)	54† (98.2%)	55 (100%)	532 (99.6%)	527 (98.7%)
**SCA**	1	1	0	0	0	0	10	10	11	11
45, X	1	1	/	/	/	/	4	4	5	5
XXY	/	/	/	/	/	/	4	4	4	4
XXX	/	/	/	/	/	/	1	1	1	1
23, X	/	/	/	/	/	/	(1)‡	1	(1)‡	1

*SCA, sex chromosome aneuploidies.

† with 1 suspected 23, X not included.

‡ suspected 23, X showing no peak for SRY and single peak for all the other markers, with ratios of AMXY/D21S11 (0.6728) and AMXY/D13S305 (0.5592) within ‘normal’ ratio ranges.

### Heterozygosity of the selected markers in the Chinese Han population

STR information and heterozygosity regarding the six sex chromosomal and two autosomal STR loci for the tested Chinese Han population are summarized in **Table 3**. The observed heterozygosity values of these STR loci ranged from 64.81% (DXS1053) to 87.37% (DXS8377).

**Table 3:** Heterozygosity and repeat numbers of the selected STR markers in the tested Chinese Han population.

STRmakers	STRloci	Repeatunit	Expectedsize/bp*	Observedsize/bp	Observedrepeat #	Observedheterozygosity** (%)
DXS1053	Xp22.2	CA	366	330–378	5∼27	64.81
DXS981	Xq13.1	TAGA	321	317–350	13∼21	76.33
DXS6809	Xq21.33	TAGA	279	251–299	27∼39	80.00
DXS1187	Xq26.2	TCTA	149	136–165	13∼20	70.42
DXS8377	Xq28	GAA	225	183–240	33∼52	87.37
X22	Xq28, Yq12	TAAAA	396	362∼450	4∼22	85.29
D21S11	21q21.1	TCTA	485	432–509	14∼33	81.08
D13S305	13q13.3	TTTC	396	392–432	41∼51	80.99

*Expected PCR product sizes are predicted according to human genome hg19 assembly.

**Heterozygosity for DXS1053, DXS981, DXS6809, DXS1187 and DXS8377 was calculated using Female samples, while heterozygosity for X22, D21S11 and D13S305 was calculated using all tested samples.

## Discussion

Although the incidence of sex chromosome aneuploidies is relatively high in the general population, there is no convenient and efficient method for diagnosis of sex chromosomal aneuploidies in prenatal diagnosis or newborn screening programs. This study has developed a rapid and reliable method which involves only simple PCR and capillary electrophoresis procedures.

With the emergence of next-generation sequencing technology, great advances have been brought to the field of non-invasive prenatal diagnosis [6]. However, samples collected by invasive methods are on-going during clinical routines and could guarantee successful and accurate diagnosis. QF-PCR on the first generation sequencing platform is much easier in sample handling, machine manipulation and data processing and thus more appropriate in clinical application.

In this study we de novo established a 10-plex QF-PCR assay for rapid diagnosis for sex chromosome aneuploidies. The markers were considerately chosen to cover regions across the whole target chromosome (**Figure 1**). In addition to X/Y-linked markers, including two sex determination genes (AMXY and SRY), five X-linked short tandem repeats (STRs; DXS1053, DXS981, DXS6809, DXS1187 and DXS8377), and one X/Y-common STR (X22), two autosomal STRs (D13S305 and D21S11) were included as internal controls for dosage quantitation of sex chromosomes. With carefully designed primers, all ten markers could be examined in one single multiplex QF-PCR assay to make experiments more convenient, cost-effective and reliable.

It has been acknowledged that the low level of polymorphism of most X-specific DNA markers has hampered the use of QF-PCR for the detection of sex chromosome aberrations [7]. Although rapid diagnostic methods for detection of sex chromosome aberrations have been developed, by either involving several X-linked markers [8] or by using one autosomal marker as internal control for quantitation of an X-linked marker [9], they have potential defects which hampered them from being used in prenatal diagnosis or as a diagnostic method for detection of sex chromosome diseases such as Turner syndrome: the former one contained not enough X-linked markers and the latter one is potentially unstable in different laboratories. By employing enough markers, with more than one internal control included in a single PCR reaction, our 10-plex QF-PCR assay possesses more advantages in experimental convenience and diagnostic reliability compared to previously reported tests.

The addition of D13S305 is useful in this 10-plex QF-PCR assay when it comes to samples of trisomy 21 or triploidies. The ratio of the overall peak area of AMXY to that of D13S305 ranges from (0.5–1.7): 1 for samples of normal karyotypes. In cases of trisomy 21, sex chromosomal markers should have normal peaks and normal ratios of AMXY peak area to D13S305, but abnormal ratios of AMXY peak area to D21S11. In cases of triploidies, the 10-plex QF-PCR assay shows abnormal peaks with normal ratios of AMXY peak area to D21S11 and D13S305. The situation is likewise when it comes to a case of trisomy 13. Only in cases of 69, XXX with single peaks for all the 9 markers (without SRY) will it be insufficiently informative, but this problematic situation is of low chance and will be solved with additional STR markers. In our practice, all suspected cases of trisomies 21, 13 or triploidies found by this 10-plex QF-PCR assay were later confirmed by using additional STR markers of chromosomes 21, 13 and 18. Nevertheless, the 10-plex QF-PCR assay was insufficiently informative in cases of mosaicism, e.g. a case of adult sex abnormality with mosaicism of 46, XX[10]/46, XY[45], and cases of structural abnormality, e.g. a case of 46X, inv(Y) revealed by conventional cytogenetic analysis.

This is the first attempt to establish a single QF-PCR test for the diagnosis of sex chromosome aneuploidies in the Chinese population. This is also the first QF-PCR test which assesses the copy number of target chromosomes by both STR peak number counting and dosage calculation in a single multiplex PCR reaction. Our results have shown that all the selected markers are of high enough heterozygosity to be employed in at least the Chinese Han population. Retrospective and prospective comparisons with conventional cytogenetic karyotyping showed that the new 10-plex QF-PCR assay has good value in clinical application at least for the Chinese Han population. Due to simple technique and data analysis, as well as easy implementation within routine clinical services, this method is of great clinical application value and could be widely applied. Our test design is also of reference value when examining the copy numbers of other chromosomes or genes.

## Materials and Methods

### Clinical sample collection

Amniotic fluid (AF), chorionic villi (CV) and cord blood samples were collected for prenatal cytogenetic analysis in the Guangzhou Women and Children’s Medical Center during November 2009 to February 2011. A total of 85 DNA samples with previous known karyotyping results, including 50 normal female/male, 20 Turner (45, X), 5 cases of 47, XXY, 5 cases of 47, XYY and 5 cases of 47, XXX, were included in the preliminary stage of the QF-PCR assay. To test the QF-PCR assay, samples undergoing prenatal karyotyping analysis were obtained, including 413 AF, 27 CV, and 39 cord blood. After collecting enough samples for cell culture by karyotypitsts, DNA was extracted from the remaining uncultured native samples and subjected to QF-PCR analysis. To increase the sample size of diseased cases, 55 CV samples from spontaneous abortions were also included. The ethics committee of Guangzhou Women and Children’s Medical Center approved of this study, and written consents were obtained from all patients involved. This study was conducted according to the principles expressed in the Declaration of Helsinki.

### DNA preparation

DNA was routinely exacted from native AF, CV, or cord blood samples using the QiaGen Mini Blood kit (Qiagen, Hilden, Germany) according to manufacturer’s instructions with two modifications: (1) remaining AF samples in 15-mL tubes provided by karyotypitsts were kept still at 4°C overnight or subjected to centrifugation at 3,000 rpm for 5 min, then 200 µL samples at the bottom were collected for DNA extraction; (2) DNA was finally eluted into deionized water and stored at −20°C for long term storage.

### Ten-plex PCR amplification and quantitative electrophoresis

Primers for well selected markers were designed using PrimerExpress (Applied Biosystems, Foster City, CA). PCR assays were performed with 0.9 unit Hot Start version Taq DNA polymerase (Takara Bio Inc., Otsu, Japan) and 10∼50 ng DNA template in a 20-µL reaction system. All the primers used in the 10-plex PCR assay are listed in **Table 1**. The cycling parameters were 95°C 5 min, (95°C 30 s, 60°C 40 s, 72°C 1 min)×25 cycles, 72°C 10 min. A loading mixture of 1 µL PCR product, 0.2 µL Genescan 500HD[ROX] size standard (Applied Biosystems) and 9 µL HiDi formamide (Applied Biosystems) was prepared and fragment analysis was performed on an ABI 3130xl automated sequencer according to the manufacturer’s instructions. GeneMapper software version 3.7 (Applied Biosystems) was applied to data analysis using the microsatellite analysis method. The advanced peak detection algorithm was used, with ranges set to be ‘full range’ and ‘all size’ to have the peaks automatically shown in full panel, with manual adjustment of the vertical range (arbitrary fluorescence unit on the vertical axis) for some samples to make visual comparison of the peaks from the same sample easier. We selected ‘Generate panel using samples’ for the software to automatically generate a panel for our selected STR markers to collect all actually observed peak sizes. Other details of peak data analysis are according to the User Guide of GeneMapper Software. Peak area and size of all markers from each sample were collected for individual diagnosis using peak number and ratio of peak areas.

### Statistical analysis

Data are shown as mean ± SD. Comparisons between two groups of data were performed by unpaired t (test. Selection of cutoff values was based on the Receiver Operating Characteristics (ROC) curve. All tests were done by GraphPad Prism 5.

## Supporting Information

Table S1 Peak size and area of representative examples.
(XLSX)
